# A Liberal Education

**Published:** 1898-02

**Authors:** 


### A Liberal Education.

Mr. Huxley says: "That man has a liberal education who has been so trained in youth that his body is the ready servant of his will, and does with will and pleasure all the work that, as a mechanism, it is capable of; whose intellect is a clear, cold, logic engine, with all its parts of equal strength and in smooth working order, ready, like the steam engine, to be turned to any kind of work, and spin the gossamers as well as forge the anchors of the mind; whose mind is stored with the knowledge of the great and fundamental truths of nature and of the laws of her operations; one who, no stunted ascetic, is full of life and fire, but whose passions are trained to come to a halt by a vigorous will, the servant of a tender conscience; who has learned to love all beauty, whether of nature or art, to hate all vileness, and to respect others as himself. Such a one, and no other, has had a liberal education."—*Medical Review of Reviews.*

